# Supplementary figures and images for: The RNA-binding KH-domain in the unique transcription factor of the malaria parasite is responsible for its transcriptional regulatory activity
Source: PLoS One. 2023 Dec 21;18(12):e0296165. doi: 10.1371/journal.pone.0296165 (PMC10734933; doi:10.1371/journal.pone.0296165)

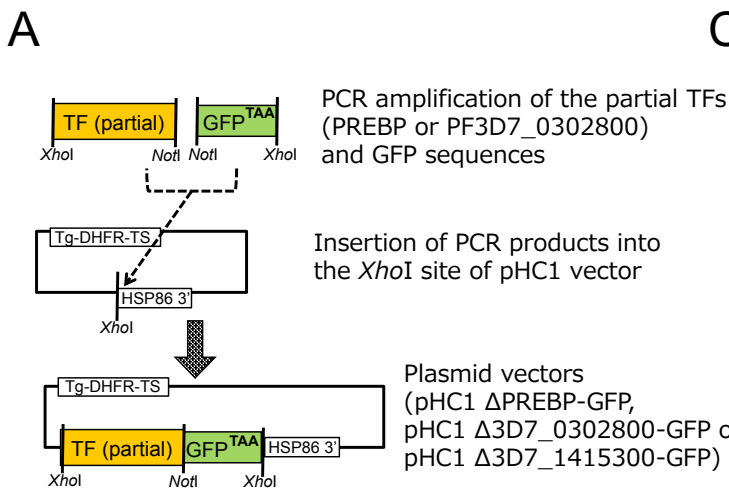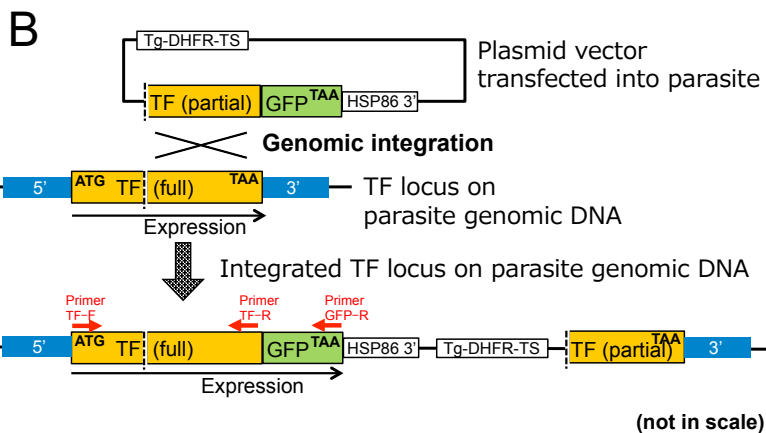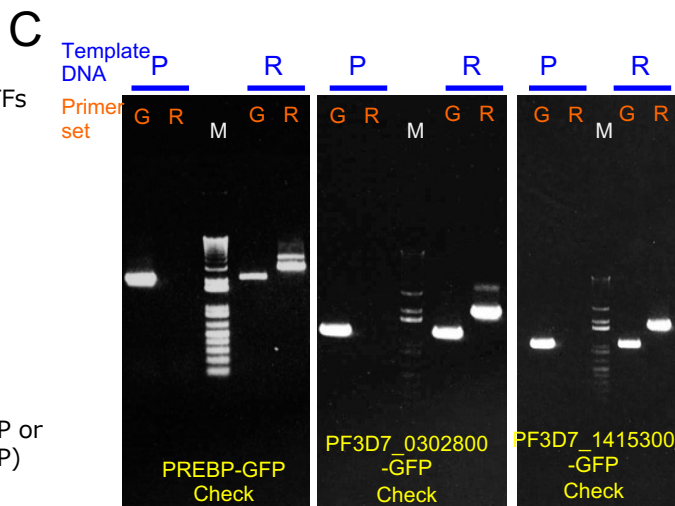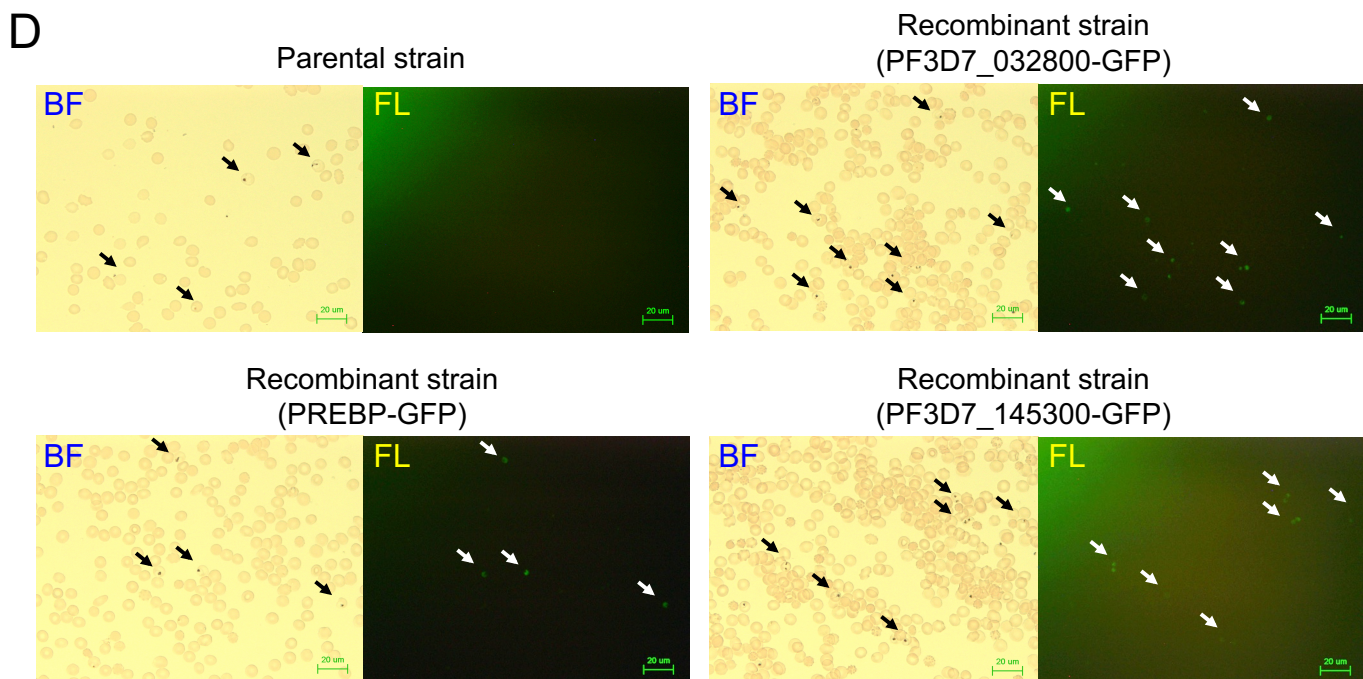

Supplement: S1 Fig — (A) The strategy for the construction of plasmid vectors used for the establishment of transgenic parasites. The partial sequence of transcription factor (TF) and the GFP sequence were amplified by PCR. The primers used for the PCR reactions were designed so that the amplicon retained XhoI and NotI sites at both ends. The PCR products were digested by XhoI and NotI and then cloned into the XhoI site of the pHC1 vector. The vector contains the T. gondii DHFR-TS gene as a resistant gene for selection by pyrimethamine. (B) Schematic illustration of the transcription factor gene (PREBP or PF3D7_032800) locus and organization following the single-crossover homologous recombination event. After recombination, TF and GFP fusion proteins were expressed under the control of the endogenous 5′ promoter. (C) PCR to check for genomic integration. Genomic DNA was extracted from the parent and each of the recombinant clonal parasite strains, and PCR was performed using them as templates. Primer sets were used to detect the ORF of each target factor gene (primer set G) and the ORF formed by the fusion of each factor gene and the GFP gene (primer set R). The primer locations are shown in the schematic illustrated in S1B Fig. The primer sequences are shown in S4B Fig. (D) Observation of the recombinant parasite strains using fluorescence microscopy. Each living parental or recombinant parasite strain was observed under a fluorescent microscope in bright (BF) and dark (FL) field. Parasites visible in the BF are indicated by black arrows, and parasites emitting GFP fluorescence visible in the DF are indicated by yellow arrows. Scale bar: 20 μm. (PDF) [file pone.0296165.s001.pdf]

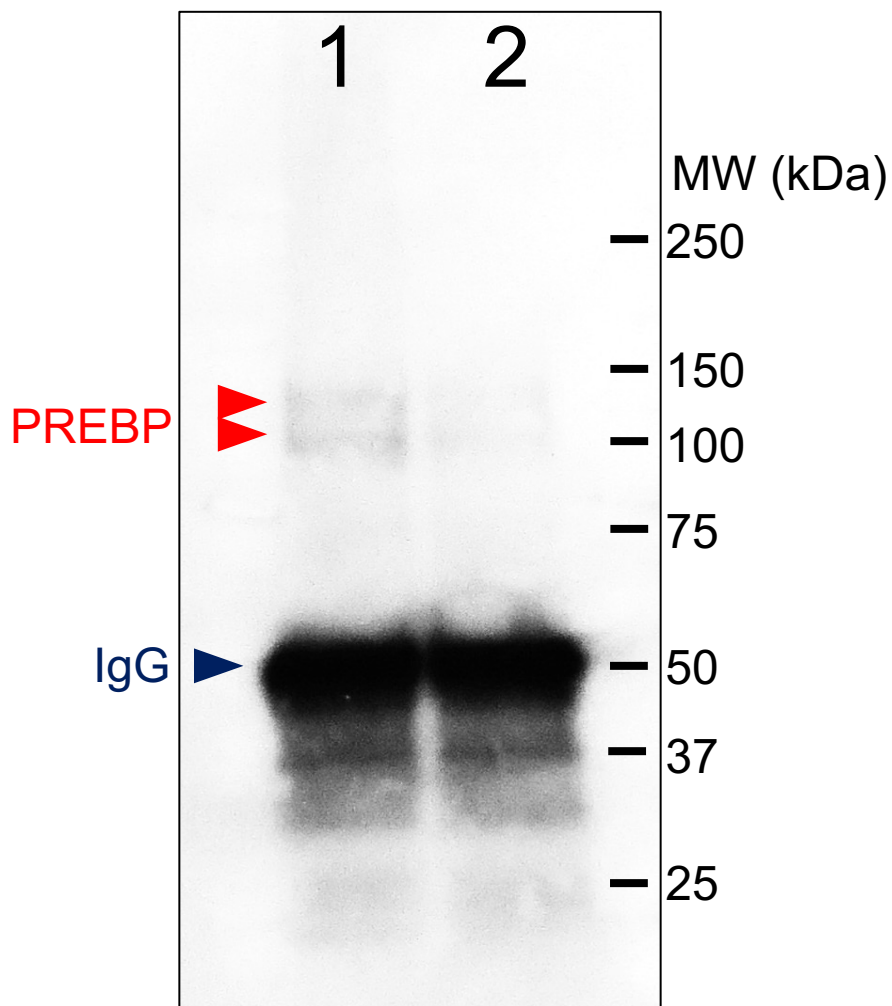

1: Precipitate by anti-PREBP antibody  
2: Precipitate by Normal rabbit IgG

Supplement: S2 Fig — The chromatonin immunoprecipitation (ChIP) assay was conducted according to the methodology outlined in the Materials and Methods section. During the immunoprecipitation step, we used a rabbit polyclonal antibody against recombinant-PREBP [18] or an equivalent amount of normal rabbit IgG for the immunoprecipitation of the chromatin sample. Subsequently, 20 μL of Dynabeads eluates containing the antigen-antibody complex were mixed with 5 μL of 5X SDS-PAGE sample buffer (comprising 5% SDS, 0.1% Bromophenol Blue, 312.5 mM Tris-HCl at pH 6.8, and 50% glycerol), and 25 mM DTT was added, followed by incubation at 100°C for 10 minutes. The mixture was then subjected to 5–20% SDS‒PAGE. Following electrophoresis, the proteins were electrophoretically transferred to polyvinylidene difluoride sheets (Immobilon; Merck-Millipore) and probed with the anti-PREBP antibody. Immune complexes were visualized using TrueBlot Anti-IgG HRP® antibody, Rabbit (Rockland Immunochemicals, Inc., Pottstown, USA), and SuperSignal™ West Dura Extended Duration Substrate (Thermo Fisher Scientific). Lane 1 corresponds to the precipitate with anti-PREBP, while Lane 2 corresponds to the precipitate with normal rabbit IgG. Molecular weight markers in kDa are indicated on the left for reference. The bands corresponding to PREBP are indicated with red arrows. (PDF) [file pone.0296165.s002.pdf]

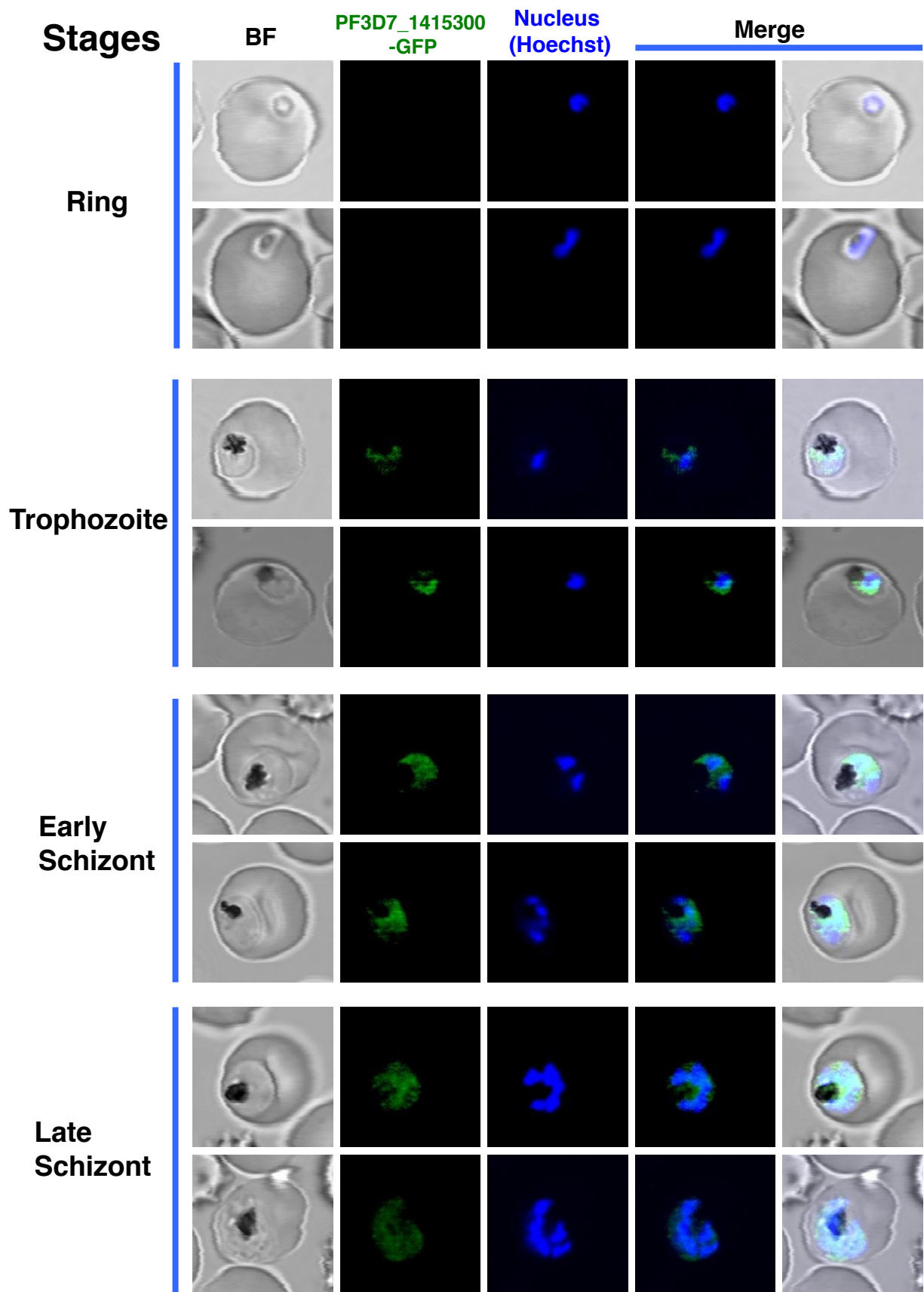

5  $\mu$ m

Supplement: S5 Fig — Living parasites that expressed the PF3D7_1415300-GFP fusion protein were observed by the same method that was used to observe the expression of PREBP-GFP. Parasites of each developmental stage were observed under 407-nm emission for the detection of Hoechst (blue) and under 488-nm emission for the detection of PF3D7_0302800-GFP (green). “BF” indicates bright-field images. “Merge” indicates merged images of Hoechst and PREBP-GFP or those of Hoechst, PF3D7_0302800-GFP, and BF. The scale bar is the same for all images. (PDF) [file pone.0296165.s005.pdf]

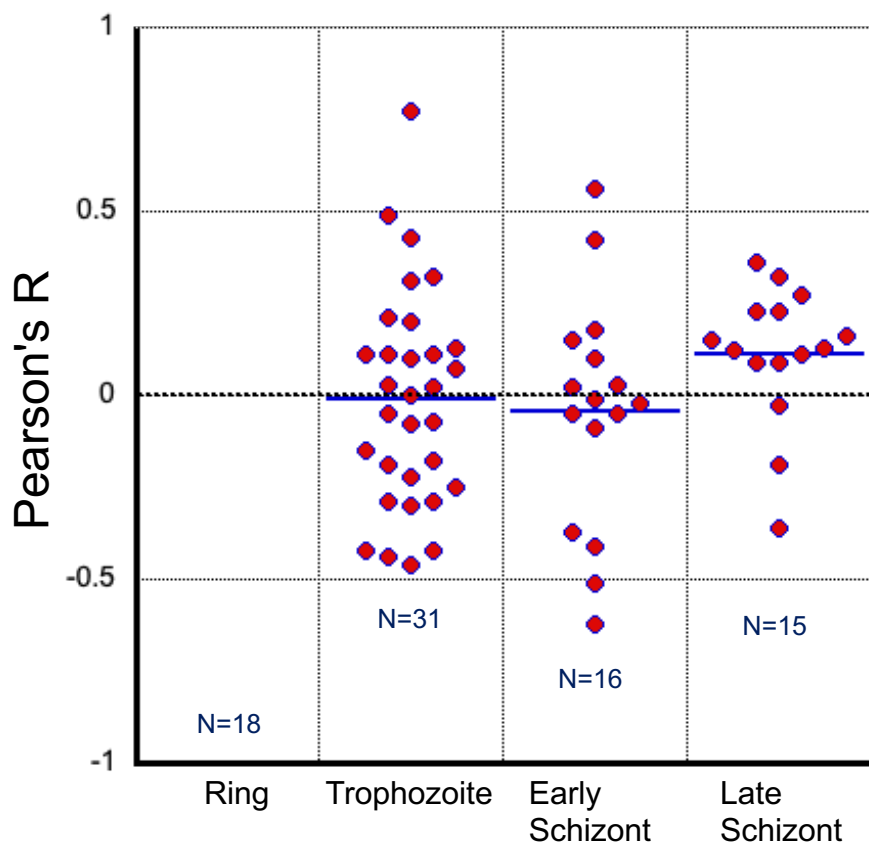

Supplement: S6 Fig — Pearson’s R value for colocalization between the nucleus, stained with Hoechst, and PF3D7_1415300-GFP was calculated. Raw data points are represented as dots on the graph, with the average indicated as a bar for each developmental stage. For all observed parasite cells in the ring stage, no GFP-derived green fluorescence could be detected, rendering the calculation of the R value impossible. The number of parasite cells analyzed in each stage of development is also displayed in the graph. (PDF) [file pone.0296165.s006.pdf]
