# Supplementary material for: The RNA-binding KH-domain in the unique transcription factor of the malaria parasite is responsible for its transcriptional regulatory activity
Source: PLoS One. 2023 Dec 21;18(12):e0296165. doi: 10.1371/journal.pone.0296165 (PMC10734933; doi:10.1371/journal.pone.0296165)

A

Group 1

	humanFBP1	PREBP	PF3D7_0302800	PF3D7_0605100	PF3D7_1415300	TGGT1_212980
Pf	PF3D7_1415300 3.E−09<br>PREBP 2.E−06<br>PF3D7_0605100 3.E−05	None	None	PF3D7_1415300 2.E−10	PF3D7_0605100 3.E−11	PF3D7_1415300 5.E−17
Tg	TGGT1_217880 9.E−08<br>TGGT1_216670 5.E−05<br>TGGT1_241170 8.E−04	TGGT1_216670 2.E−12<br>TGGT1_246190 7.E−07<br>TGGT1_462965 5.E−04	TGGT1_226320 1.E−35	None	TGGT1_217880 2.E−87<br>TGGT1_241170 5.E−25<br>TGGT1_2129980 7.E−27<br>TGGT1_320080 4.E−07	TGGT1_217880 1.E−19<br>TGGT1_241170 5.E−08<br>TGGT1_320080 3.E−05
Cp	cgd1_1280 7.E−04	cgd7_1890 1.E−11	cgd4_130 5.E−06	None	cgd7_720 8.E−15	cgd7_720 1.E−33

	TGGT1_216670	TGGT1_217880	cgd4_130	cgd7_720	cgd7_1890
Pf	PREBP 3.E−13	PF3D7_1415300 5.E−91<br>PF3D7_0605100 2.E−07	PF3D7_0510100 5.E−33<br>PF3D7_032800 3.E−04	PF3D7_1415300 1.E−14	PREBP 2.E−11
Tg	None	TGGT1_241170 2.E−21<br>TGGT1_212980 8.E−18	TGGT1_314860 9.E−05	TGGT1_212980 2.E−32<br>TGGT1_217880 2.E−11	TGGT1_216670 3.E−14
Cp	cgd7_1890 1.E−15	cgd7_720 3.E−11	None	None	None

Group 2

	PF3D7_0623600	TGGT1_314860	cgd4_1210
Pf	None	PF3D7_0623600 5.E−131	PF3D7_0623600 8.E−134
Tg	TGGT1_314860 1.E−133	None	TGGT1_314860 9.E−110
Cp	cgd4_1210 5.E−122	cgd4_1210 2.E−113<br>cgd4_130 5.E−05	None

Group 3

	TGGT1_235930	cgd1_1280
Pf	None	None
Tg	TGGT1_209210 5.E−29	TGGT1_235930 4.E−95<br>TGGT1_209210 3.E−31
Cp	cgd1_1280 2.E−95	None

Orphans

	TGGT1_209210	TGGT1_237550	TGGT1_241170	TGGT1_271250	TGGT1_320080	cgd2_2940
Pf	None	None	PF3D7_1415300 5.E−24<br>PF3D7_0605100 3.E−05<br>PF3D7_0215600 8.E−05<br>PREBP 2.E−04	None	PF3D7_1415300 3.E−07	None
Tg	TGGT1_235930 2.E−28	None	TGGT1_217880 8.E−21	None	TGGT1_212980 9.E−05	None
Cp	cgd1_1280 6.E−31	None	None	None	None	None

B

Putative homologs for Group 1 proteins

Putative homologs for Group 2 proteins

Putative homologs for Group 3 proteins

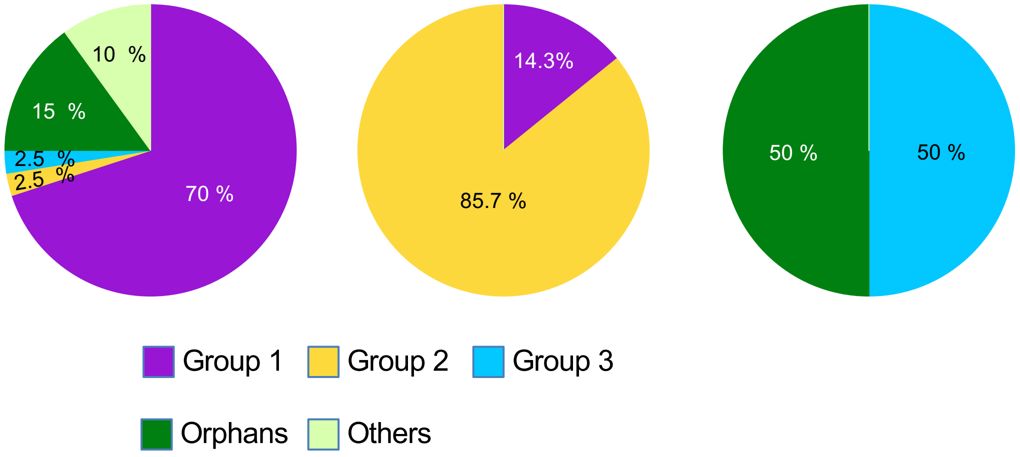

Supplement: S3 Fig — (A)The genome database for P. falciparum (Pf), T. gondii (Tg), and C. parvum (Cp) was searched using the BLASTP program with each candidate protein. The proteins used for each BLASTP search are indicated at the top of each table. The genome databases used for each search are shown on the left side of each table, and homologs suggested by each BLASTP search are indicated inside tables with e-values. Putative homologs with e-values of <1 × 10^3^ are indicated. The protein itself, which was subjected to the search and indicated as a homolog, is not included in the table. Protein belonging to group 1 (Fig 5) is indicated with a purple box. Proteins indicated with yellow, light blue, and green boxes belong to group2, group 3, and orphans, respectively. “None” means that no homolog with a significant e-value was found. Proteins indicated with a light-green box are proteins without hypothetical KH domains. (B) The pie charts depict the distribution of homolog types identified through BLASTP searches for each candidate protein. The percentages representing each homolog type in the graph were calculated using the data presented in (A). Different homolog categories, including those belonging to group 1, group 2, group 3, orphans, and others (proteins not included in candidate proteins with KH domains), are represented by distinct colors: purple, yellow, light blue, green, and light green, respectively. (PDF) [file pone.0296165.s003.pdf]
