# Supplementary material for: The RNA-binding KH-domain in the unique transcription factor of the malaria parasite is responsible for its transcriptional regulatory activity
Source: PLoS One. 2023 Dec 21;18(12):e0296165. doi: 10.1371/journal.pone.0296165 (PMC10734933; doi:10.1371/journal.pone.0296165)
Supplement: S4 Fig — In order to express the fusion protein of PF3D7_0605100 and GFP for P. falciparum, the plasmid shown in S1A Fig was introduced into the cultured parasites via electroporation. Screening of genetically modified parasites was performed through cultivation in the presence of 0.1 μM pyrimethamine. Under these conditions, for the three strains introduced the plasmids for the fusion proteins of PREBP, PF3D7_0302800, and PF3D7_1415300 along with GFP, it took less than three months to observe the disappearance of pyrimethamine-induced parasite death, with an increase in growth speed. This suggested that the introduced plasmids containing drug resistance genes integrated into the genome. However, for the plasmid for the PF3D7_0605100-GFP fusion gene introduced parasites, pyrimethamine-induced parasite death was still observed even after three months from the transfection, indicating that the drug resistance gene did not integrate into the genome and remained episomal. At this point, a limiting dilution was performed with the goal of cloning all four recombinant parasites. For the PREBP, PF3D7_0302800, and PF3D7_1415300, stable parasite lines expressing the fusion proteins with GFP were successfully established (S1 Fig). However, for PF3D7_0605100, pyrimethamine-induced parasite death continued even after limiting dilution. Subsequently, RNA was extracted from all strains, and cDNA was synthesized for each, following the methods described in a previous report [16]. RT-PCR was conducted to confirm the expression of the respective genes fused with GFP. (A) PCR to confirm the expression of fusion gene. PCR was performed using cDNA prepared from RNA extracted from each parasite strain as a template. Primer sets were used to detect the cDNA of each target gene (primer set E) and the cDNA transcribed from the fusion mRNA of each factor gene and the GFP gene (primer set F). The primer sets and locations are the same as those shown in the primer sets G and R in S1B Fig. For PREBP, PF [file pone.0296165.s004.pdf]

A

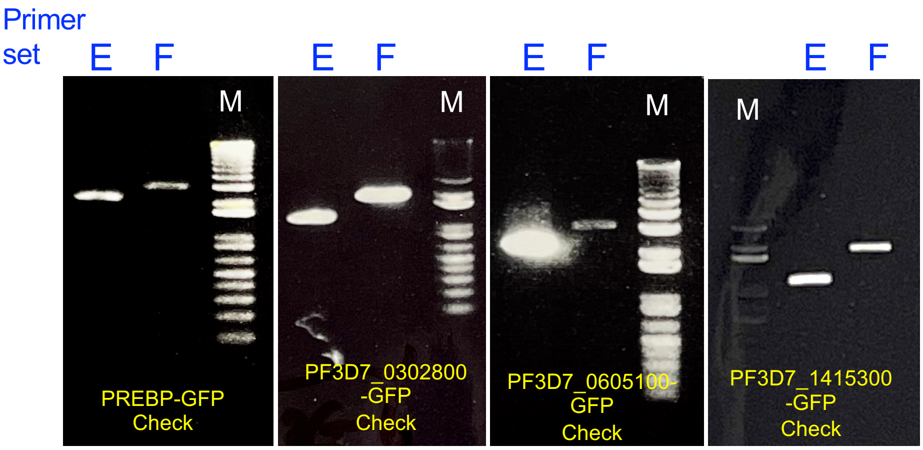

Expected size of each PCR product

Target gene	primer set E	primer set F
PREBP-GFP fusion	2.1 kb	2.8 kb
PF3D7_0302800-GFP fusion	1.2 kb	1.9 kb
PF3D7_0605100-GFP fusion	2.2 kb	2.9 kb
PF3D7_1415300-GFP fusion	1.0 kb	1.7 kb

Primer set
E: each TF-F & TF-R (for check of endogenous or fusion mRNA)
F: each TF-F & GFP-R (for check of fusion mRNA)
M: 1kb ladder

B

Primer sequences used for RT-PCR

PREBP F	GATTATGCCATACCAACTGACACA
PREBP-R	AAGGAAAAAAGCGGCCGCAATTTTCATTTGATTGTTTTTCTTGTTCCT
PF3D7_0302800-F	ATGTCTATAAGAAATAGAGATAAGAATTATATGA
PF3D7_0302800-R	AAGGAAAAAAGCGGCCGCGGTTATTACCATATGCACCA
PF3D7_060510-F	ATGATAAAACAACAAAAAAGATCATATGCACT
PF3D7_060510-R	AAGGAAAAAAGCGGCCGCTGGATTGTATCATAAGAATCGC
PF3D7_1415300-F	ATGGTTGAAATTAATAAAGGAGACCATCGA
PF3D7_1415300-R	AAGGAAAAAAGCGGCCGCATGGATCACATTTAATTTGCATAGT
GFP-R	GCGCTCGAGTTATTTGTATAGTTCATCCATGCCA
