# Supplementary material for: The RNA-binding KH-domain in the unique transcription factor of the malaria parasite is responsible for its transcriptional regulatory activity
Source: PLoS One. 2023 Dec 21;18(12):e0296165. doi: 10.1371/journal.pone.0296165 (PMC10734933; doi:10.1371/journal.pone.0296165)
Supplement: S1 Table — (DOCX) [file pone.0296165.s007.docx]

**S1 Table. List of primers used for constructing the plasmids employed in this study.**

| **Primers for the construction of p1-10R PREBP ΔC2 andΔC3** | |  | |
| --- | --- | --- | --- |
| Name | Sequence (5'-3') | | |
| NotI-HSP86 3'F | AAGGAAAAAAGCGGCCGCTTATATAATATATTTATGTACTCGCAATG | | |
| ΔC2F | AAGGAAAAAAGCGGCCGCTTAGAGGCTAGCGTAATCCGGAACATCGTATGGGTATTTTAATATTTCATTTAATTTTTCTTCTGCCT | | |
| ΔC3F | AAGGAAAAAAGCGGCCGCTTAGAGGCTAGCGTAATCCGGAACATCGTATGGGTATTAAATCCTCTAGCTA | | |
| **Primers for the construction of p1-10R PREBPΔN1, ΔN2 and ΔN3** | | | |
| Name | Sequence (5'-3') | | |
| NotI-FLAGR | AAGGAAAAAAGCGGCCGCCTTGTC GTCATCGTCT | | |
| ΔN1F | AAGGAAAAAAGCGGCCGCAGATGATATTGAACAATTTATTTC | | |
| ΔN2F | AAGGAAAAAAGCGGCCGCCAAAAATATTAAACTCGATTCCA | | |
| ΔN3F | AAGGAAAAAAGCGGCCGCTAGATCCAAAGAAGA AATGTCA | | |
| **Primers for the construction of p1-10R PREBPΔC1** | | |  |
| Name | Sequence (5'-3') | | |
| FLAG-PREBP-F | GCGCTCGAGAGGACTACAAAGACGATGACGACAAGATGGGAAGGAAAGCTACCA | | |
| ΔC1-R | GCGCTCGAGTTAGAGGCTAGCGTAATCCGGAACATCGTATGGGTATGACATTTCTTCTTTGGATC | | |
| **Primers for the construction of pHC1-ΔPREBP-GFP and pHC1-ΔPF3D7_0302800-GFP** | | | |
| Name | Sequence (5'-3') | | |
| Xho-PREBP-F | GCGCTCGAGGAAAGAAATAGAAACCAACACAAACACA | | |
| Not-PREBP-R | AAGGAAAAAAGCGGCCGCAATTTTCATTTGATTGTTTTTCTTGTTCCT | | |
| Xho-3D7_0302800-F | GCGCTCGAGGAGCACATGATATAAGGATATTAGCTGA | | |
| Not-3D7_0302800-R | AAGGAAAAAAGCGGCCGCGGTTATTACCATATGCACCA | | |
| PSPT-GFP-F | GTTCTGTTCCAGGGGCCCATGTCTAAAGGAGAAGAACTTTTCA | | |
| Xho-GFP-R | GCGCTCGAGTTATTTGTATAGTTCATCCATGCCA | | |
| Not-PSPT | AAGGAAAAAAGCGGCCGCCTGGAAGTTCTGTTCGAGGGGCCCAT | | |

Restriction enzyme target sequences are highlighted in red.
