# Supplementary material for: The RNA-binding KH-domain in the unique transcription factor of the malaria parasite is responsible for its transcriptional regulatory activity
Source: PLoS One. 2023 Dec 21;18(12):e0296165. doi: 10.1371/journal.pone.0296165 (PMC10734933; doi:10.1371/journal.pone.0296165)
Supplement: S2 Table — (DOCX) [file pone.0296165.s008.docx]

**S2 Table. Candidate proteins with KH domains identified in searches of PlasmoDB, ToxoDB, and CryptoDB for the bioinformatic analysis.**

| **From PlasmoDB release 28** | |  |
| --- | --- | --- |
| Gene ID | Annotation | Reason for exclusion from the analysis |
| PF3D7_1469300 | small subunit rRNA processing KH domain protein, putative | rRNA processing related protein |
| PF3D7_0208200 | KRR1 small subunit processome component, putative (KRR1) | rRNA processing related protein |
| PF3D7_0302800 | conserved Plasmodium protein, unknown function |  |
| PF3D7_0510100 | Conserved Plasmodium protein, unknown function | No KH domain was found by SMART3 |
| PF3D7_0605100 | RNA-binding protein, putative |  |
| PF3D7_0623600 | transcription or splicing factor-like protein, putative |  |
| PF3D7_1011800 | PRE-binding protein (PREBP) |  |
| PF3D7_1359600 | Conserved Plasmodium protein, unknown function | No KH domain was found by SMART3 |
| PF3D7_1415300 | RNA-binding protein, Nova-1, putative |  |
| PF3D7_1435800 | GTPase Era, putative (ERA) | No KH domain was found by SMART3 |
| PF3D7_1465900 | 40S ribosomal protein S3 | rRNA processing related protein |
| **From ToxoDB release 28** | |  |
| Gene ID | Annotation | Reason for exclusion from the analysis |
| TGGT1_226320 | Hypothetical protein | No KH domain was found by SMART3 |
| TGGT1_212980 | Hypothetical protein |  |
| TGGT1_217880 | Putative RNA-binding protein Nova-1 |  |
| TGGT1_241170 | Hypothetical protein |  |
| TGGT1_216670 | FUSE-binding protein 2, KH-type splicing regulatory protein |  |
| TGGT1_220490 | putative pre-rRNA-processing protein PNO1 | rRNA processing related protein |
| TGGT1_209210 | Hypothetical protein |  |
| TGGT1_235930 | Domain K- type RNA binding proteins family protein |  |
| TGGT1_246190 | Hypothetical protein | No KH domain was found by SMART3 |
| TGGT1_320080 | Hypothetical protein |  |
| TGGT1_314860 | Zinc knuckle domain-containing protein |  |
| TGGT1_237550 | Hypothetical protein |  |
| TGGT1_271250 | Hypothetical protein |  |
| TGGT1_309960A | Hypothetical protein | No KH domain was found by SMART3 |
| **From CryptoDB release 28** | |  |
| Gene ID | Annotation | Reason for exclusion from the analysis |
| cgd4_130 | RRM domain and KH domain protein (SPAC30D11.14-like KH) |  |
| cgd4_1210 | Ms15p |  |
| cgd7_720 | PASILLA splice variant 3-like 2KH domains, transmembrane domain at C-terminus |  |
| cgd7_1890 | KH domain protein |  |
| cgd8_1650 | PNO1 | rRNA processing related protein |
| cgd1_1280 | Domain KOG1676, K-homology type RNA binding proteins |  |
| cgd2_2940 | Conserved hypothetical protein |  |

The proteins included in the subsequent analysis are highlighted in yellow.
