# Supplementary material for: The RNA-binding KH-domain in the unique transcription factor of the malaria parasite is responsible for its transcriptional regulatory activity
Source: PLoS One. 2023 Dec 21;18(12):e0296165. doi: 10.1371/journal.pone.0296165 (PMC10734933; doi:10.1371/journal.pone.0296165)
Supplement: S3 Table — Identity (%), Positives (%), Gaps (%) and E-value indicate alignment results through BLAST when comparing each homolog with the query protein. (DOCX) [file pone.0296165.s009.docx]

**S3 Table. List of homologs of Group 1 proteins preserved in *Plasmodium* species.**

| **Query: the sequense of PF3D7_0302800** | | |  |  |  |  |  |
| --- | --- | --- | --- | --- | --- | --- | --- |
| Species | ID | gene product | protein length | Identities | Positives | Gaps | E-value |
| *P. berghei* | PF3D7_0302800 | RNA-binding protein, putative | 419 | 419/419 (100%) | 419/419 (100%) | 0/419 (0%) | 0.00E+00 |
| *P. chabaudi* | PBANKA_0401400 | RNA-binding protein, putative | 474 | 246/412 (60%) | 304/412 (74%) | 29/412 (7%) | 2.00E-143 |
| *P. chabaudi* | PCHAS_0402300 | RNA-binding protein, putative | 466 | 228/382 (60%) | 278/382 (73%) | 15/382 (4%) | 1.00E-130 |
| *P. cynomolgi* | PCYB_084640 | hypothetical protein | 388 | 184/366 (50%) | 238/366 (65%) | 39/366 (11%) | 1.00E-109 |
| *P. reichenowi* | PRCDC_0302100 | RNA-binding protein, putative | 418 | 410/421 (97%) | 413/421 (98%) | 5/421 (1%) | 0.00E+00 |
| *P. vivax* | PVX_119265 | hypothetical protein, conserved | 517 | 228/421 (54%) | 282/421 (67%) | 49/421 (12%) | 2.00E-141 |
| *P. yoelii* | PY17X_0403600 | RNA-binding protein, putative | 473 | 230/383 (60%) | 284/383 (74%) | 22/383 (6%) | 6.00E-135 |
|  |  |  |  |  |  |  |  |
| **Query: the sequense of PF3D7_0605100** | | |  |  |  |  |  |
| Species | ID | gene product | protein length | Identities | Positives | Gaps | E-value |
| *P. falciparum* | PF3D7_0605100 | KH domain-containing protein, putative | 755 | 755/755 (100%) | 755/755 (100%) | 0/755 (0%) | 0.00E+00 |
| *P. berghei* | PBANKA_0103900 | KH domain-containing protein, putative | 735 | 498/780 (64%) | 584/780 (75%) | 70/780 (9%) | 0.00E+00 |
| *P. chabaudi* | PCHAS_0104500 | KH domain-containing protein, putative | 724 | 506/766 (66%) | 584/766 (76%) | 53/766 (7%) | 0.00E+00 |
| *P. cynomolgi* | PCYB_115130 | RNA binding protein | 782 | 526/811 (65%) | 603/811 (74%) | 85/811 (10%) | 0.00E+00 |
| *P. reichenowi* | PRCDC_0603700 | KH domain-containing protein, putative | 756 | 746/761 (98%) | 750/761 (99%) | 11/761 (1%) | 0.00E+00 |
| *P. vivax* | PVX_113420 | RNA-binding protein, putative | 810 | 517/853 (61%) | 592/853 (69%) | 141/853 (17%) | 0.00E+00 |
| *P. yoelii* | PY17X_0105400 | KH domain-containing protein, putative | 742 | 178/243 (73%) | 201/243 (83%) | 11/243 (5%) | 6.00E-102 |
|  |  |  |  |  |  |  |  |
| **Query: the sequense of PF3D7_1415300** | | |  |  |  |  |  |
| Species | ID | gene product | protein length | Identities | Positives | Gaps | E-value |
| *P. falciparum* | PF3D7_1415300 | RNA-binding protein Nova-1, putative | 337 | 337/337 (100%) | 337/337 (100%) | 0/337 (0%) | 0.00E+00 |
| *P. berghei* | PBANKA_1027400 | RNA-binding protein Nova-1, putative | 337 | 227/323 (70%) | 271/323 (84%) | 3/323 (4%) | 1.00E-158 |
| *P. chabaudi* | PCHAS_1028200 | RNA-binding protein Nova-1, putative | 341 | 234/325 (72%) | 281/325 (86%) | 15/325 (5%) | 2.00E-163 |
| *P. cynomolgi* | PCYB_134140 | RNA-binding protein Nova-1, putative | 278 | 216/280 (77%) | 248/280 (89%) | 2/280 (1%) | 4.00E-154 |
| *P. reichenowi* | PRCDC_1414600 | RNA-binding protein Nova-1, putative | 337 | 334/336 (99%) | 334/336 (99%), | 0/336 (0%) | 0.00E+00 |
| *P. vivax* | PVX_085685 | RNA-binding protein Nova-1, putative | 335 | 266/336 (79%) | 303/336 (90%) | 2/336 (1%) | 0.00E+00 |
| *P. yoelii* | PY17X_1029800 | RNA-binding protein Nova-1, putative | 338 | 222/322 (69%) | 271/322 (84%) | 12/322 (4%) | 1.00E-155 |

Identity (%), Positives (%), Gaps (%) and E-value indicate alignment results through BLAST when comparing each homolog with the query protein.
