# Supplementary material for: The RNA-binding KH-domain in the unique transcription factor of the malaria parasite is responsible for its transcriptional regulatory activity
Source: PLoS One. 2023 Dec 21;18(12):e0296165. doi: 10.1371/journal.pone.0296165 (PMC10734933; doi:10.1371/journal.pone.0296165)
Supplement: Attachment — Submitted filename: Response to Revieweres 231207.docx [file pone.0296165.s011.docx]

7 December 2023

Dear Editor,

We would like to express our gratitude to you and the reviewer for the thorough review of our manuscript. We are pleased to learn that our manuscript is deemed suitable for publication in PLOS ONE following minor grammatical corrections.

Kindly confirm that all the points listed below have been addressed in the revised manuscript in accordance with your suggestions:

LN 16: “The PREBP had been shown” should be “PREBP has been shown..”

LN 18: “The PREBP…” should be “PREBP…”

LN 27-28: “Bioinformatic analysis suggested…” should be “Bioinformatic analysis revealed…”

LN 75: “…PREBP was expected…” should be “…PREBP was predicted from…”

LN 84: “The finding of PREBP suggested…” should be “ These findings suggest…”

LN 95: “unknown” should be “putative”

LN 371: “(see Fig 4A)” should be “(Fig 4A)”

LN 409-410: “…organisms, unknown KH-domain containing proteins might act as unknown transcription factors” should be “…organisms, KH-domain containing proteins might act as transcription factors.”

LN 611-612: “…in the Apicomplexan…” should be “…in Apicomplexan…”

Regarding the reviewer's suggestion to modify the title, we believe in the significance of the factor with RNA-binding being involved in transcriptional regulation through DNA. Therefore, we kindly request your understanding in retaining the current title.

We have thoroughly reviewed the reference list and can confirm that it is complete, correct, and that no paper has been retracted.

Thank you very much.

Sincerely yours,

Kanako Komaki-Yasuda

Department of Tropical Medicine and Malaria Research Institute

National Center for Global Health and Medicine

1-21-1 Toyama, Shinjuku, Tokyo 162-8655, Japan

Tel: +81 3 3202 7181 (ext. 2878)

Fax: +81 3 3202 7364

E-mail: komaki@ri.ncgm.go.jp
